# Shared topics on the experience of people with haemophilia living in the UK and the USA and the influence of individual and contextual variables: Results from the HERO qualitative study

**DOI:** 10.3402/qhw.v10.28915

**Published:** 2015-11-16

**Authors:** Laura Palareti, Silvia Potì, Frederica Cassis, Francesca Emiliani, Davide Matino, Alfonso Iorio

**Affiliations:** 1 Department of Education Studies, University of Bologna, Bologna, Italy; 2 Hemophilia Center, University of São Paulo Faculty of Medicine Clinics Hospital, São Paulo, Brazil; 3 Health Information Research Unit, Department of Clinical Epidemiology and Biostatistics, McMaster University, Hamilton, ON, Canada

**Keywords:** Haemophilia, patient perspective, thematic analysis, psychosocial, cross-cultural

## Abstract

The study illuminates the subjective experience of haemophilia in people who took part in the Haemophilia Experience, Results and Opportunities (HERO) initiative, a quali-quantitative research program aimed at exploring psychosocial issues concerning this illness around the world. Applying a bottom-up analytic process with the help of software for textual data, we investigated 19 interviews in order to describe the core themes and the latent factors of speech, to explore the role of different variables in shaping the participants’ illness experiences. The five themes detected are feeling different from others, body pain, acquisition of knowledge and resources, family history, and integration of care practices in everyday life. We illustrate how nationality, age, family situation, the use of prophylaxis or on-demand treatment, and the presence of human immunodeficiency virus or hepatitis C virus affect the experience of our participants in different ways. Findings are used to bring insights on research, clinical practice, and psychosocial support.

Haemophilia, a rare, inherited, chronic condition, is caused by a partial lack of the factors responsible for blood clotting (factors VIII or IX). Its transmission is recessive and sex chromosome linked; therefore, it manifests only in men, while women are carriers (generally asymptomatic). The illness can also be caused by a spontaneous genetic mutation; therefore, some people with haemophilia (PWH) do not have a family history of this disease.

Haemophilia is characterized by repeated bleeding episodes in the joints (typically knees, ankles, and elbows) and soft tissue (skin, muscles), which cause haematomas and hemarthrosis. Without adequate treatment, individuals can experience swelling, stiffness, functional limitations, inflammation, pain, and a progressive degenerative process. Internal bleeding can also occur, and there is a risk for serious life-threatening complications, such as bleeding in the brain. Haemorrhages can follow physical trauma, or be spontaneous, depending on the severity of the condition.

Therapy procedures—to be continued throughout one's life—consist of the intravenous administration of the deficient clotting factor. This therapy can be administered in an emergency room, in a haemophilia centre, or at home through self-infusions. It can take two forms—on-demand or prophylaxis. In the first case, the clotting factor is administered after every bleeding episode, requiring timely intervention and considerable skill in promptly evaluating the symptoms. In prophylactic treatment, the factor is administered regularly, two or three times per week, even in the absence of bleeding episodes. This second method reduces the anxiety and the alarm surrounding physical trauma, but can be quite invasive, thus entailing a significant everyday psychological and organizational burden for affected individuals and their care givers (Brigati & Emiliani, 2013). Moreover, therapies are expensive and, given that it is a chronic condition, they represent a significant economic cost for the community—in countries where health care is guaranteed by the state—or for the individuals—in countries where treatment is only possible through private insurance.

Nowadays, at least in developed countries, haemophilia is a very different disease than it was a few generations ago (Gringeri et al., 2004). Medical advances, like prophylaxis and the availability of safe clotting factor, have reduced life-threatening events and have prevented chronic complications. We must mention that in the early 1980s many PWH were infected with the human immunodeficiency virus (HIV) or hepatitis C virus (HCV) because of transfusions with infected blood. Acquired immunodeficiency syndrome (AIDS) caused high mortality rates as well as social stigmatization and marginalization of haemophiliacs. Older generations of PWH may still have the HIV and/or HCV virus. Even if at present the risk of contamination with infected blood has technically been eliminated, researchers have observed that the fear of contamination and the stigma persist in social relationships (Barlow, Stapley, & Ellard, 2007), also because this illness is inevitably linked to symbolic and taboo elements, such as blood and needles (Markova, Linell, Grossen, & Salazar Orvig, 2007; Potì, 2013). Therefore, PWH and their families often have to deal with an array of psychosocial challenges and emotions (e.g., shame, guilt, fear, and anxiety), and the illness can have a significant impact on their quality of life regarding relationships and the management of school, work, or leisure activities (Beaton, Neal, & Lee, 2005; Cassis, Emiliani, Pasi, Palareti, & Iorio, 2012; Cassis et al., 2014; Tejero Pérez, 2005).

Some authors have investigated the impact of haemophilia from a bio-medical and individual psychological perspective, measuring dimensions such as self-esteem, stress, anxiety levels, and depression among PWH or care givers (Basu, Chowdhury, & Mitra, 2010; Ghanizadeh & Baligh-Jahromi, 2009; Kyngas & Rissanen, 2001; Plug et al., 2008). In recent years, the increasing attention to the perceived impact of illness led to several studies on the quality of life of PWH, with the creation of disease- and age-specific instruments (Berg et al., 2015; Bradley et al., 2006; Szende et al., 2003). Nevertheless a systematic review of methodologies and findings on the psychosocial aspects of haemophilia has evidenced that research in this area is still limited, based on questionnaire techniques with little or no qualitative information, and that there is a lack of data on the haemophilia life cycle, along with data from developing countries (Cassis, Querol, Forsyth, & Iorio, 2012). Although useful for providing quantitative information, this literature can be limited in grasping the mechanisms and process that lead people toward different kind of outcomes and to variations in the levels of well-being or social integration (Emiliani, Palareti, & Melotti, 2010).

In an era where the psychosocial care of chronic conditions is increasingly recommended (Holland, Watson, & Dunn, 2011), some authors claim the need for further and international studies that take into account the subjective perspectives of people in the variety of contexts and circumstances they have to face (Breakey, Blanchette, & Bolton-Maggs, 2010; Cassis, et al., 2012; Glozah, 2015).

In the present paper, we took advantage of old data gathered by the multinational Haemophilia Experience, Results and Opportunities (HERO) program to run a qualitative study that, overcoming some of the gaps evidenced in the literature, explores the psychosocial construction of meanings of living with haemophilia.

## Method

The HERO program (www.herostudy.org) was established in 2009 as a multimethod initiative aimed at broadening the understanding of the psychosocial issues associated with living with haemophilia. After a literature review, in the first phase, 150 face-to-face interviews with PWH, parents, and health care professionals (HCPs) in seven countries were collected (Algeria, Brazil, France, Germany, Italy, United Kingdom, and United States) and their explicit content was used to elucidate the key psychosocial issues and to prioritize areas for the following quantitative assessment (Forsyth et al., 2014). Then two online surveys (one for adult PWH and one for parents of children with haemophilia) were developed and conducted in 10 countries. The 1236 questionnaires gathered represent the largest multicountry data set yet gathered, including demographic, treatment, and psychosocial information at the same time.

In this paper, a different approach to the analysis of the interviews of PWH is applied in order to deepen the subjective meanings of the illness experience, in particular cultural and social contexts (Markova et al., 2007).

Our specific goals were to identify the core themes shared by our participants and to explore the way in which the national context, the life-cycle stage (age and family situation), and the clinical condition (type of treatment, and presence of HIV or HCV virus infection) affect their illness experience.

The face-to-face interviews were performed in 2010 by a specialist health care research agency. They generally took place in the homes of PWH and lasted at least 60 min each. The interview grid (see Supplementary file) was prepared by two of the authors, in collaboration with the research agency, to explore the following issues: the first awareness of haemophilia; the meaning of growing up with haemophilia; current issues related to living with haemophilia; haemophilia treatment; support received; and hopes for the future. The interviewer encouraged participants to talk about their own personal experiences through non-directive prompts and a list of open-ended questions. At the end of the interview, they were also asked to complete a questionnaire on demographic and social data. All of the interviews were audio recorded with the patients’ informed consent and transcribed verbatim. The relevant ethical boards of the countries involved approved the study.

### Participants

We purposely selected only interviews of PWH living in the United States (USA) and the United Kingdom (UK), because they are characterized by the same linguistic matrix (English-speaking first-world countries), but have opposite health care systems. Until recently the health care system in the USA was mainly based on private insurance, whereas in the UK there is a national health system (Lelli, 2013).

Participants were contacted through patients’ associations and received a letter from the HERO board and the research agency that presented the research aims and methods. Because the general aim of the qualitative phase of this HERO project was to explore different PWH's points of view, all adult men with haemophilia A or B who were willing to participate in an in-depth face-to-face interview were enrolled in the study. Nine PWH were interviewed in the UK and 10 in the USA, divided according to the variables illustrated in Table I. Although it was a convenience sample, the Fisher's exact test confirmed the independence between nationality and all the other variables considered, indicating that the interviewees are equally heterogeneous in the two countries.

**Table I:** Characteristics of the sample according to the variables.

Country	UK	USA	Total
Therapy			
Prophylaxis	6	5	11
On demand	1	3	4
Both	2	2	4
Age group			
1 (18–30)	4	4	8
2 (31–50)	3	6	9
3 (51–70)	2	0	2
HIV and/or HCV			
Yes	2	6	8
No	7	4	11
Family situation			
Single	6	6	12
Dating	2	1	3
Married	1	0	1
Married with Children	0	3	3
Total	9	10	19

### Data analysis

A bottom-up thematic analysis was performed on the 19 interviews. The inductive approach was chosen as particularly well suited to bring out subjective experiences that are socially constructed and context-bounded (Guba & Lincoln, 1994; Holloway & Wheeler, 2013; Vaismoradi, Turunen, & Bondas, 2013). In particular, we analysed the transcripts using the “Thematic analysis of elementary context” from T-Lab software (Lancia, 2012).

The software is used to study vocabulary and co-occurrence matrices to identify shared themes or issues associated with the topics being researched, which allows for the exploration of the latent content (Braun & Clarke, 2006) of the entire data set. In the thematic analysis, the software examines the text of all the interviews considered as a single set of data, identifying the interviewees’ lexical choices, and performing cluster and correspondence analysis (Caputo, 2014; Emiliani, Bertocchi, Potì, & Palareti, 2011; Montali, Monica, Riva, & Cipriani, 2011). Beyond the statistical analysis, the researcher is highly involved while using the software, from the preparation of the data set, to the interpretation of the statistical outputs. Therefore, he/she must be very familiar with the interview texts and the research topic. In the present study, two of the authors prepared the data set (e.g., lemmatization and disambiguation of terms, choice of the keywords) and conducted the T-Lab analysis together. Subsequently, a third author analysed the results of the statistical outputs, and together the authors redefined the interpretations and labelled the data.

Of the transcribed interviews, we choose to import only the participants’ answers into T-Lab, obtaining a data corpus of 101 pages (font 12, single-spaced) for a total of 76,326 words (before the lemmatization and creation of the dictionary). Each interview was labelled according to the set of variables selected for this study:

- country of the interviewee: USA; UK
- family situation: single; dating; married; married with children
- type of therapy: on-demand; prophylaxis
- presence/absence of HIV and/or HCV virus: Yes (H yes); No (H no)
- age group: age 1 (18–30); age 2 (31–50); age 3 (51–70)

The Thematic Analysis of Elementary Context produced the following outputs:

- the clusters, described through a set of keywords tended to be associated according to their decreasing X2 value. The rationale is that a set of co-occurring words marks a specific thematic content. Therefore, sentences having a certain set of co-occurring words in common share the thematic content marked by such a set. The association of words in each cluster allows the researcher to reconstruct the links that arise on a psychological level in the respondents, recognizing how the group of participants cognitively and emotionally conceptualizes that particular theme. Each cluster has a different weight according to the percentage of text (sentences) that includes with respect to the entire textual corpus;
- the main factors (the axes of the factorial space, generally 2 or 3) that describe the joint behaviour of groups of lemmas and that can be interpreted as significant semantic dimensions expressed by the conversation. Each polarity is formed by different keywords that most often co-occur in the same parts of the text;
- a graphic representation of the position of the variables and clusters on the factorial space. The closeness between a cluster and a variable indicates that the theme is particularly relevant for participants that share that variable.

## Results

**Figure 1:**
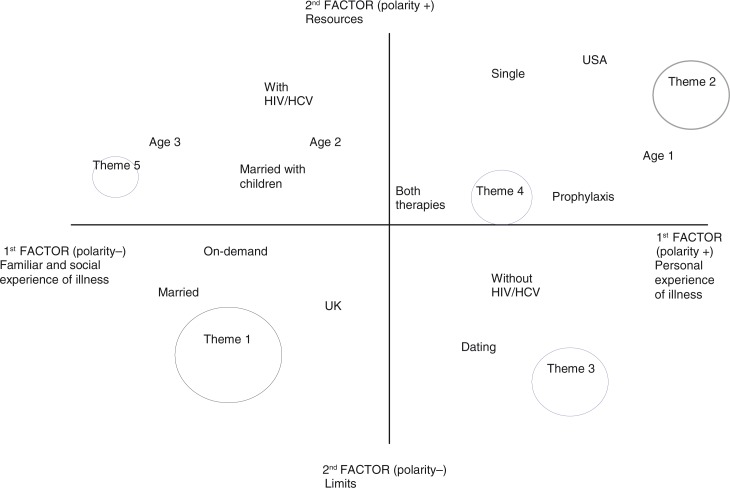
Factorial plane, with labelled factorial polarities, and positions of themes and variables.

The analysis identified five clusters, that is, the themes, located in the factorial space illustrated in Figure 1. We labelled the polarities of each factor interpreting the words that characterize them. In this way we can see that the first factor represents in its left polarity the familiar and social experience of illness. On the right side is evoked instead the personal experience of haemophilia in the everyday life.

The negative pole of the second factor is centred on pain, distress, and limits, whereas the positive one is focused on resources for coping with illness and, in particular, on the support system.

### The themes

Below you will find the description of each theme along with a table that lists the chain of words that characterizes it (in the order of decreasing associative value), and some illustrative sentences. Each table also indicates the weight of the cluster according to the percentage of text included.

1. Growing up and feeling different from others, even within one's own family (Table II).

**Table II:** Cluster 1 “Growing up and feeling different from others, even within own family.”

Weight	31.92% (406 sentences)
Keywords and χ^2^ a	my (31.05), condition (29.33), parents (24.60), HIV (20.58), young (18.97), severity (18.76), play (17.83), think (15.94), haemophilia (15.89), brother (15.74), early (15.41), hospital (15.36), understand (15.28), teenager (15.27), blood (14.75), mom (14.27), children (14.11), wrong (13.56), talk about (11.38), together (10.14), sure (10.12), age (9.86), support (9.33), spend (8.66), impact (8.65), unique (8.55), frame (8.55), remember (8.12), time (8.02), college (7.68), school (7.60), grow up (7.60), the others (7.22), matter (6.87), life (6.82), different (6.82), mum (6.62), dad (6.51), football (5.98), careful (5.87), feel (5.86), day (5.71), family (5.64), take care (5.57), listen (5.57), old (5.38), cotton wool (5.34), share (5.27), expect (5.27), failed (5.05), worst (5.05) annoyance (5.05), behind (5.03), effects (5.03), kick (5.03), be able (4.47), primary school (4.41) anxiety (4.14)
Extracts	«I think that as I am the younger brother and as I had haemophilia, I think that my parents put a lot more emphasis on taking care of me when I was younger, whereas my brother probably needed some of that attention and maybe he missed out on that (US, Age group 1, Prophylaxis, Single, without HIV/HCV)». «One teacher poked me in the back and I got covered in bruises. My mum went and spoke to the school. Communication was poor when I was a child. The haemophilia society was helpful. Speaking with other parents who were bleak about the condition, they said we would be crippled by 30. Professor said not to treat us much differently (UK, Age group 2, Prophylaxis, Single, without HIV/HCV)». «I have a feeling indirectly it has impacted our relationship. Now obviously we are older and we talk well and everything, but when we were younger if he would punch me as any brother would do to any brother and then my parents would go: “he has got haemophilia, are you crazy?” (US, Age group 1, Prophylaxis, Single, without HIV/HCV)».

a The words are listed in the order of decreasing associative value (χ^2^ in brackets).

This is the biggest cluster and is placed on the third quadrant of the factorial plane. It focuses on the meaning that being a haemophiliac has over time, as the condition plays a role in an individual's growth within the family. Respondents have the impression of being on the wrong side; they also feel as like they are in a glass bubble, wrapped in cotton wool, untouchable, different from the others. The impact of haemophilia at school, in relationships with others (especially with healthy brothers and sisters), and in sport, with the risk of becoming socially isolated, is evident in this theme. This issue is associated with boredom, expectations, failure, and anxiety. It is very interesting that in the development of the theme, HIV is one of the first words, suggesting that the historical contagion that affected the haemophilia community has been a relevant worry in our participants’ lives.

2. Cognitive strategies to manage the illness and find resources (Table III).

**Table III:** Cluster 2 “Cognitive strategies to manage the illness and find resources.”

Weight	23.58% (300 sentences)
Keywords and χ^2^ a	call (46.08), read (43.20), clinic (39.58), explain (32.86), on-demand (27.90), ask (27.55), nurse (27.22), study (26.80), camp (25.19), factor (24.82), infusion (23.26), work (22.54), learn (19.29), consultant (18.80), treatment (15.41), doctor (15.33), research (14.86), local (1.86), F8 (14.86), dose (14.09), prophylaxis (13.70), source (13.00), treatment centre (12.69), N7 (12.04), emergency room (12.04), needs (11.94), treat (11.42), good (11.41), answer (11.16), patients (10.84), product (10.62), insurance (10.35), pay (9.87), medication (9.71), company (9.33), recombinant (9.33), inhibitor (9.02), physician (8.93), early treatment (8.86), how to do (8.84), break (8.84), money (8.23), summer (7.43), aids (7.41), social worker (7.40), question (7.40), needs (6.94), support group (6.62), country (6.20), crisis (6.20), relationship (5.94), calculate (5.88), on line (5.88), Sunday (5.88), national (5.88), Europe (5.88), Community (5.26), know how (4.48)
Extracts	«I see the social worker, the nurse, the haematologist, the dentist, the physiotherapist, the orthopaedic surgeon and somebody else […]. At the 3 o'clock in the morning when you need to infuse and you can't get into a vein, you have to call the treatment centre (US, Age group 2, Prophylaxis, Single, with HIV/HCV)». «It has been explained to me pretty thoroughly and also I do a lot of my own research online just to read what other people are saying. Just to actually read research per se. From all those sources, I definitely know pretty much as much as my doctor (US, Age group 1, On-demand, Dating, with HIV/HCV)». «Actually they [HCPs] didn't have to do a lot of explaining. I read the patient insert because of my medical background I understood exactly what it was saying. I knew how to calculate my own dose. The little things that I didn't know were … I understood that you needed to take the medication as soon as possible, but there are certain things, such as: why? (US, Age group 2, Prophylaxis, Single, with HIV/HCV)».

a The words are listed in the order of decreasing associative value (χ^2^ in brackets).

This big cluster is located on the first quadrant of the plane and highlights the effort to learn how to self-manage the disease and its treatment, generally seen as a break in life, especially in leisure time or on holidays. PWH look for scientific explanations and new information on how to deal pragmatically with their condition, expressing a responsible use of resources and know-how. Different HCPs are evoked and represent a crucial point of reference whose support nourishes hope. The evaluation of what it means to be a person with haemophilia in different countries in terms of opportunities and cost of treatment surfaced.

3. Illness as disability and stressor event (Table IV).

**Table IV:** Cluster 3 “Illness as disability and stressor event.”

Weight	20.60% (262 sentences)
Keywords and χ^2^ a	pain (75.29), bleed (67.55), knee (66.51), joint (64.11), bad (57.64), big (37.,91), week (31.90), muscle (29.15), injury (26.32), damage (26.32), heal (23.23), ankles (18.43), happen (17.60), tolerance (15.46), codeine (15.46), car (15.04), target (15.04), month (14.78), calf (14.56), series (14.50), health (14.40), hit (14.28), walk (14.04), worse (13.85), disability (13.46), physical (12.76), difficult (12.76), arthritic (11.76), recover (11.76), allowance (10.83), limit (10.61), problem (10.59), mobility (9.65), frustration (8.87), day (8.82), fit (8.64), minute (8.64), serious (8.56), worry (8.45), deal with (8.15), catch up (7.82), swollen (7.82), stress (7.81), painkillers (7.37), bend (7.26), feel down (7.26), wheelchair (7.07), broke (6.77), daily (6.77), elbow (6.63), disappointment (6.46), bruise (6.40), feet (5.74), legs (5.45), treatment at home (5.24), routine (4.76), morphine (4.76), pills (4.76)
Extracts	«At 21, I had a really bad bleed, and it became a target joint, and I just had bleed after bleed after bleed. It was no longer a healthy joint, not by far […]. And so that was pretty bad, and when I had the series of bleeds I couldn't walk (US, Age group 1, On-demand, Dating, with HIV/HCV)». « […] the doctor let me go on to home to do the treatment. He didn't give me any painkillers unless it was absolutely necessary. The high doses of morphine they give me frightened me. When I was 11, I had a painful bleed in the Soleus muscle and I tried morphine for a few days, but I felt awful after that (UK, Age group 3, On-demand, Single, without HIV/HCV)». «I am unemployed, limited by joint damage. I am 24 years old: not an age to be sat like this doing nothing. Just had a left ankle fixed, right ankle fused, right knee replaced, looking to have elbow surgery (UK, Age group 1, Prophylaxis, Dating, without HIV/HCV)».

a The words are listed in the order of decreasing associative value (χ^2^ in brackets).

This cluster is located on the fourth quadrant and covers 20% of the text. The core is the body, described through a detailed review of its injured and vulnerable parts. Feelings of depression and frustration emerged, related to the limitations and pain caused by the illness in everyday life. In this theme, we find explicit references to medical aids, especially to drugs used to provide pain relief. Haemophilia appears to be a stressful and painful event that marks time (not just the everyday, but also weeks and months), creating new routines experienced with difficulty.

4. Learn to manage emotions and action toward normality (Table V).

**Table V:** Cluster 4 “Learn to manage emotions and action toward normality.”

Weight	15.80% (201 sentences)
Keywords and χ^2^ a	end up (49.65), come back (25.16), mean (24.42), bottle (20.66), comfortable (18.18), hard (17.89), learn (17.53), able (16.76), home (15.93), little (15.93), fine (14.84), water (14.79), walk (14.65), night (14.28), feelings (10.72), computer (10.56), high (10.24), shot (9.09), limp (9.04), neighbour (9.04), treatment centre (8.65), allow (8.65), leave (8.33), protective (8.33), high school (7.78), hot (7.37), actual (7.37), feel good (7.37), syringe (7.37), smaller (7.37), skate (7.37), wheelchair (7.09), social (7.07), mind (7.06), vein (6.68), summer (6.68), know how (6.68), legs (6.41), important (6.34), myself (6.33), normal (6.22), responsibility (6.09), cost (5.58), cope (5.58), crutches (5.29), receive (5.29), God (5.29), start (5.14), needle (4.46), activities (4.46), build (4.43), each other (4.43), emotions (4.31), insurance (4.21), relationship (4.18), attention (3.87), office (3.87), regimen (3.87)
Extracts	«After that, once I started getting my actual vein that was another huge success point. It was such a huge turnaround that I was able to do my medicine by myself (US, Age group 2, On-demand, Married with children, with HIV/HCV)». «Everyone was like a little more mature, and I went to a pretty good high school, so maybe people didn't have an obnoxious outlook on diseases. They were just all pretty normal about it. So that was fine (US, Age group 1, On-demand, Dating, with HIV/HCV)». «Come back on my feet when I was probably about 16 getting out of the wheelchair that's a huge one. I had intracranial bleeding I had to learn how to walk and talk and everything again. Just the recovery from head bleeds just learning the motor functions again (US, Age group 2, On-demand, Married with children, with HIV/HCV)».

a The words are listed in the order of decreasing associative value (χ^2^ in brackets).

This smaller cluster is positioned in the first quadrant, together with Cluster 2, and refers to the processing of meanings and practices designed to integrate the illness and its treatment into the everyday, with the aim of creating a sustainable routine in the search for normality. The cluster gives a picture of people that are involved in many social contexts and have to cope with emotions and feelings without trying to ignore them. Both clusters in this quadrant place emphasis on learning to manage haemophilia. Yet, whereas Cluster 2 is full of references to medical aspects, Cluster 4 describes the existential dimension of the illness within everyday life contexts.

5. A tremendous mortal game: the discovery of genetic and family history (Table VI).

**Table VI:** Cluster 5 “A tremendous mortal game: the discovery of genetic and family history.”

Weight	8,10% (103 sentences)
Keywords and χ^2^ a	mother (159.57), carrier (127.15), family (113.92), father (104.62), sister (103.17), uncle (83.48), mom (82.96), side (69.01), daughter (68.20), boy (51.40), grandparents (46.80), dad (37.44), haemophilic (34.87), siblings (34.87), brother (31.99), passed away (28.58), death (26.67), scared (25.56), raise (24.13), India (23.78), generation (22.75), cousin (18.99), diagnosis (18.99), family history (18.17), son (18.17), children (17.15), test (16.92), sick (14.22), pissed (14.22), nobody (11.91), expect (11.91), born (11.67), long time (11.42), share (11.06), divorce (9.46), loss (9.46), ready (9.46), blame (9.35), job company (7.75), treat (7.75), bled (7.75), close (to someone) (7.32), genetic (6.86), persistent (6.68), negative (6.49), found out (6.49), grow up (6.43), hell (5.48), tremendous (5.15), possibility (4.65), crazy (4.65), hide (4.65), lucky (4.65), girl (4.65), rare (3.96), horrible (3.96), funny (3.96), game (3.96)
Extracts	«As far as I know, my great grandfather on my mother's side had haemophilia. That's pretty much as far as history of haemophilia goes in my family, at least as far as we know. I guess there have been daughters, daughters carriers and then a boy. I was the lucky one (US, Age group 1, On-demand, Dating, with HIV/HCV)». «Then when they came back later and gave me the carrier testing, then they found out that my mom was a carrier. So then they come back and tried to check with the uncles in the family and because at that time the older generations, the African American, they wouldn't talk about certain things. There were a couple of uncles who passed away, but none knew of what or why (US, Age group 2, Prophylaxis, Single, with HIV/HCV)». «My younger brother is deceased. My others to sisters are still alive and functional. My mom is still alive, my dad is deceased. I am divorced. I have no children. I am the only person in my immediate family that has it. I had three nephews who were haemophiliacs, and they are all deceased (US, Age group 2, Prophylaxis, Single, with HIV/HCV)».

a The words are listed in the order of decreasing associative value (χ^2^ in brackets).

This small, but significant, theme on the second quadrant focuses on the search for origins, even back to previous generations and countries of origin, in order to probe the genealogic tree. In this theme, illness is experienced as a form of death. Together with science and genetics, randomness and misfortune are considered in a game described as horrible, like Russian roulette. The emotions that characterize this theme are fear, anger, and blame. Loss is evoked in different aspects of life, such as work or romantic relationships.

### Exploring differences related to individual and contextual variables

If we analyse the position of structural variables on the factorial plane, we can see that the four modalities describing the family situation of our participants are distributed on the four different quadrants. In particular, more than others, married people represent haemophilia as a possible limit to relationships, whereas those married with children are more concerned with the issue of genetic and family history. Single people emphasize the importance of coping strategies in everyday life, and seek social support and resources through HCPs, whereas dating individuals stress the physical problems and the limits of their body, expressing concern and sufferance.

Referring to age, we can observe that as people grow older, they move from a primary interest in personal day-to-day experience, to being engaged with the issue of genealogy, family history, and death. Older interviewees are also near the variable “with HIV/HCV,” because of the years in which they have contracted the viruses, whereas the young are near the variable “prophylaxis,” reflecting the changes in health care.

As expected, patients affected by HIV/HCV are rather close to the image of haemophilia as a mortal game but mainly underline the role played by the health care system. Persons with haemophilia without these viruses are more interested in the limits in their everyday lives, mostly worried about the other disease complications. Note that the issue of HIV, elicited in the first thematic group, is transversal to people with and without infection. Confirming the results of other studies (Pasqual Marsettin et al., 1995, 1998), this finding indicates that fear of contagion and the derived social stigma still have a strong emotional impact also for seronegative PWH.

On-demand therapy is more closely associated to an image of haemophilia that creates worries and feelings of being different from others starting from infancy, whereas prophylaxis is related to a more normalized life style, where emotions are elaborated and the illness is better managed.

Finally, the US respondents stressed the aspects linked to the existing support systems more, such as the relationship with HCPs or the cost of health insurance, whereas people in the UK considered the issues of pain, diversity, and disability in the everyday life more. Although the specific characteristics of participants may have produced this outcome by chance, it seems consistent with the differences in the two health care systems to us. Probably, in fact, where people pay directly for care (USA), PWH are more demanding toward professionals, paying attention to their relationships with them and taking notice of the quality of care received. Instead, in UK, where the health care system is public, the relationship with professionals is not in the forefront and interviewees remain focused on their personal experience of sickness and its impact on other relational contexts, such as family, school, or work.

## Discussion

### Strengths and limitations of the study

Haemophilia is a rare disease and this study is based on a small easily accessible sample of people who were interviewed in 2010. Therefore, any attempt to extend or generalize its results must be done with caution. However, because the criterion for judging the soundness of a qualitative study is not its generalizability but rather the transferability of the results (Guba & Lincoln, 1994), we believe our 19 respondents represent a valuable source of information with which to explore the psychosocial construction of meaning in living with this chronic condition. Moreover, the length and quality of the interviews were ideal for elaboration with the T-Lab analytic software. We consider the use of T-Lab a further strength because it allows one to preserve the richness and ideographical elements of the subjective experience and, at the same time, be accurate and rigorous (Lancia, 2007, 2012) in analysing the data.

In order to achieve our goal, we choose a method focused on how concepts (words) are associated in the whole data set, not the interpretation of the explicit content of each interview. The associations, detected through statistical analyses, have allowed for the emersion of latent aspects of the material in terms of shared content (number and characteristics of the themes), in terms of semantic and symbolic dimensions expressed in the conversation (the factors), and in terms of the contribution given by each of the investigated variables to the final result (the position of the variables in the map).

The use of quantitative statistics does not reduce the qualitative nature of the study. Therefore, the co-researchers were in constant dialogue to expand, check, or correct their viewpoints throughout the whole process.

Lincoln and Guba state that the aim of trustworthiness in a qualitative research is to support the argument that the inquiry's findings are “worth paying attention to” (1985, p. 290). We believe that this study offers insights that can help professionals to develop more effective practices that are culturally relevant and respondent to patients’ needs. In fact, simply providing patients with information or behavioural prescriptions in order to elicit attitudes that promote treatment compliance has not proved sufficient. This approach implies that people are blank pages on which one can write the best conduct, and that people will always make the best choices. Conversely, it is important to understand in what knowledge system, and with which symbolic and emotional meanings, the new information will be integrated and implemented.

The indication of the details of the statistical outputs in the tables (the exact sequence of words characteristic of each theme) was not only chosen for transparency but also as an invitation to the reader to consider the affective and cognitive meanings expressed by the interviewees without the mediation of, and inevitably reduction by, the researchers.

### Research and clinical implications

Regarding additional research connected with the HERO initiative, it would be interesting to explore the interviews given to parents and HCPs in the UK and the USA in order to assess similarities and differences in the relevance given to certain topics, like pain or relationships between patients and HCPs. Moreover, the same thematic analysis could be performed on the interviews of PWH living in developing nations (Algeria and Brazil, where access to haemophilia care and treatment is more limited) and in European non-English speaking countries (France, Germany, and Italy), in order to further explore the role that sociocultural variables have in modifying the experience of this illness. Finally, it will be important to use the quantitative data of the last HERO phase, to check whether some issues evidenced in this study (e.g., concern over the cost of the disease in the USA, or the experience of physical suffering and marginalization in the UK) are confirmed in a larger sample.

We believe that these results can also be useful in clinical practice, because a deeper understanding of patients’ experience of haemophilia can help the HCPs to consider the subjective, developmental, and cultural aspects that often remain unstated within interactions, thus fostering therapeutic alliance with them (Khair, 2013; Sorrentino, Guglielmetti, Gilardi, & Marsilio, 2015). In particular, this explorative study suggests the following considerations:

- counselling for PWH should address social and family relations including the fear of stigma, the fear of rejection, lack of confidence, and communication with carers who tend to overprotect PWH;
- HCPs should take into account that the fear of HIV/HCV infection through blood can still be present, even in rich developed countries;
- the interpersonal relationships with HCPs, economic concerns, and the perceived costs of the illness should also be addressed, particularly in the context of private health care systems, as in the USA;
- psychosocial interventions and HCPs should take into account the issue of pain management to help PWH to manage frustration and avoid becoming psychologically dependent on painkillers (Elander & Barry, 2003; Montali, et al., 2011); and
- finally, it is important to analyse the explanations that people give themselves about being ill, identifying models that refer to faith, fate, and/or genetics. As we can see from our data, this issue is even more relevant for men with children, because these models not only affect their reproductive choices, but also their relationship to new generations. The psychosocial support provided to this target group could improve the process of making sense of their illness as well as communication with affected or carrier children.

## Conclusions

The main purpose of the present study was to explore and highlight shared aspects of the haemophilia experience for certain PWHs in the USA and the UK. We replaced the objective view of the phenomenon with the subjective perspectives of people who engage daily in the process of constructing symbolic meanings of the illness and utilize them in their own social, personal, and cultural contexts. If we consider haemophilia to be “an illness to care for” and not a “disease to cure” (Brigati & Emiliani, 2013), a number of psychosocial issues emerge that we should be aware of, particularly regarding the emotional and cognitive aspects of that particular experience. If we consider the factorial plane as a whole, focusing on the positions of the different clusters and the meanings that they express, we find a coherent representation of the feelings, worries, and resources these patients display in facing their psychosocial challenges. The picture reminds us how suffering from a chronic haemorrhagic illness is related to a life history steeped in pain.

This study does not only reveal the shared themes but also illustrates some differences linked to personal and contextual variables such as country, family status, age, and HIV or HCV status.

We trust that this composite representation can offer diverse suggestions for planning and developing clinical and psychosocial interventions, and thereby improve comprehensive care of haemophilia.

## Supplementary Material

Shared topics on the experience of people with haemophilia living in the UK and the USA and the influence of individual and contextual variables: Results from the HERO qualitative study
