# Supplementary material for: Shared topics on the experience of people with haemophilia living in the UK and the USA and the influence of individual and contextual variables: Results from the HERO qualitative study
Source: Int J Qual Stud Health Well-being. 2015 Nov 16;10:10.3402/qhw.v10.28915. doi: 10.3402/qhw.v10.28915 (PMC4649019; doi:10.3402/qhw.v10.28915)
Supplement: Shared topics on the experience of people with haemophilia living in the UK and the USA and the influence of individual and contextual variables: Results from the HERO qualitative study [file QHW-10-28915-s001.pdf]

PSYCHOSOCIAL ASPECTS OF HAEMOPHILIA
DISCUSSION GUIDE –PEOPLE WITH HAEMOPHILIA

I. INTRODUCTION (2 mins)

II. WARM UP

A. Please tell me about yourself and your family

- Family members, brothers (older/younger) sisters (older/younger), children, parents. Haemophilia amongst family members – e.g. uncle or brother or nephew of PWH may have haemophilia;
- Involvement in college, work, social activities.

III. FIRST AWARENESS OF HAEMOPHILIA

"First I want to talk about when you were a young child and first became aware that you had haemophilia or aware that you had a condition that needed treatment."

A. When did you first become conscious of the fact that you had haemophilia? How did this awareness develop?

B. At what age did you first understand what having haemophilia meant?

C. What involvement did your parents have in telling you about haemophilia? When did they do this? How did they handle this?

D. Who else did you talk to when you were a child to understand better about having haemophilia? What were you told? How helpful was each of the individuals who spoke to you about the condition? What methods of communication were used that were most helpful and least helpful?

E. What were you told that was not helpful or even harmful?

F. How did you feel at the time of this growing awareness?

G. ACCORDING TO FAMILY SITUATION: If your sister had a son with haemophilia, what advice would you give when your nephew first becomes aware of the condition?

- What would you suggest should be done differently compared to what happened to you?
- What would you deliberately suggest should be handled the same way?

III. GROWING UP WITH HAEMOPHILIA

"Now moving through your childhood up to the point where you finished your education…."

A. What was it like to grow up with haemophilia? How did haemophilia affect you at various stages of your childhood? (e.g. when starting school for the first time, when moving to senior/high school, when leaving school/moving to further education etc.).

B. *Show the PWH the timeline graph where the 'x' axis (horizontal) represents age; the left hand side is 'birth/0 years' and the right hand side the age when they finished school or further education. Mark off the years along the axis using an appropriate scale. Ask the respondent to write in the upper half the various points of their life that have been great successes/highs despite them having haemophilia/where they have overcome the effects of haemophilia and draw in the bottom half those points in their life that have been disappointments and crisis points because of haemophilia. Ask the respondent to use the vertical axis to indicate the extent of the 'high' of the success points and the 'depth' of the lows.*

AIM FOR THE RESPONDENT TO GIVE THREE OF FOUR KEY LOW POINTS. FOR THE MAIN LOW POINTS/DISAPPOINTMENTS/CRISES DISCUSS: (MAKE SURE THE LOW POINT AND WHEN IT OCCURRED IS IDENTIFIED ON TAPE).

õ Why was this point a particular disappointment or low point? Tell me more about it……

õ What was the impact of the haemophilia at this time? How did it affect your life? How did you feel at this time? How did it affect the rest of the family?

õ How did you get through this point? What did you do to overcome/alleviate this disappointment/low point?

õ Who did you turn to for help and advice? Who provided the most useful help, advice and support?

õ Does this disappointment/low point still have an ongoing impact?

õ What would you do differently if you were faced with the same situation again?

õ What would you want other people to do differently if you were faced with the same situation again?

AFTER DISCUSSING EACH LOW POINT ASK:

C. Has your ability to cope with these low points and disappointments ever been an issue? Have you ever experienced burnout at these times when you felt overwhelmed, helpless, disillusioned or completely worn out? When? How did you overcome this?

FOR THE MAIN POINTS OF SUCCESS/HIGH POINTS DISCUSS:

õ Why was this point a particular success or high point? Tell me more about it…..

D. At what point did you take over the responsibility for your own treatment? How did this process occur? How could it have been handled better?

E. How did your parents handle your haemophilia? How did it impact them? How well did your parents understand your perspective? What difficulties did you have with them? Do you think they were over-protective – in what ways? Were you anxious about telling them about bleeds or troubling them (being a burden)?

F. ACCORDING TO FAMILY SITUATION: How has haemophilia affected other members of the family? Grandparents? Brother/sisters? How has it impacted your relationship with them? How did it impact the relationship of your brothers/sisters with your parents?

G. To sum up this section, what would you say it is like to be a child or young adult growing up with haemophilia?

IV. LIVING WITH HAEMOPHILIA NOW

A. How has haemophilia impacted your life as an adult? How does it impact your day to day routines?

õ What are the easy things to cope with?

õ What are the difficult things to cope with?

õ How does it limit what you do? What can you not do because of your haemophilia but really want to do?

õ How much does the pain of a bleed or concerns about pain trouble you? How often do you experience pain from bleeds? How do you manage this? Do you take analgesics/pain relievers?

B. Which of the pictures on this board best sums up your situation now? SHOW BOARD A (PWH). Why did you choose this picture? What is the person in the picture thinking? Tell me about the similarities between the person in this picture and you. Tell me more about this…

C. Who else knows that you have haemophilia? What is their reaction and level of support and understanding?

D. Who do you meet with regularly who do not know that you have haemophilia (boss/colleagues/friends/girlfriends etc.)?

õ How do you decide who to tell and not to tell?

õ When is it appropriate to tell someone and what do you tell them?

õ Are there any issues or difficulties caused by people who do not know that you have haemophilia but somehow become aware that 'something is different' about you? Please tell me more about this.

E. Do you know any other people with haemophilia? What is their situation? How do you know them? What have you learnt from them? What have they learnt from you?

F. Do you know any other people who live with different chronic conditions? What is their situation? Do you feel worse or better off than these people? Are there similarities? What are the differences?

V. TREATMENT OF HAEMOPHILIA

A. What medical treatment are you currently using to treat your haemophilia? Is this used on-demand/when a bleed occurs, or is it used as prophylaxis?

B. How well has the medication been explained in terms of when and how to use it? Why do you say that?

C. Who has given these explanations? How well did they do? What could they have done better?

D. What medical treatment have you taken in the past to treat your haemophilia? Why was a change made? How was this change made? Who initiated the change?

E. Has a switch been made from prophylaxis (as a child) to on-demand (as an adult)? Tell me more about this? When did this occur? How was this managed? What impact did it have on your haemophilia?

F. Do you always follow the treatment guidelines of when and how much factor to use? Why? Why not? When do you do something different?

G. Apart from a physician, which other healthcare professional (e.g. nurse, psychologist, physiotherapist) and/or other support professionals (e.g. social worker, patient organisation) have been involved in managing your haemophilia and its consequences? What have they contributed? How well did they do? What could they have done better? How closely do you follow their advice/guidance? PROBE SPECIFICALLY ON PHYSIOTHERAPY.

VII. SUPPORT RECEIVED

A. What ongoing support and advice are you given now?

B. Have there been certain points in your adult life where support and help is more necessary than at other times? What are the crisis points for someone with haemophilia when additional support and guidance would be welcome?

C. REVIEW SUPPORT RECEIVED/OFFERED BY EACH INDIVIDUAL OR ORGANISATION INVOLVED:

õ Has the support been adequate/inadequate? In what ways?

õ What has been the best and the worst support that you have received?

õ Which single person has been most helpful? Why? Which single person has been most understanding? Why?

õ What additional support has been required, but has not been forthcoming? What difference would it make if this support was available?

D. What ongoing support and advice are you given now? Who from?

E. Are there times (or have there been times in the past) when you feel you are given too much support and advice or 'overprotected'; when you feel you just want to be left alone? Tell me about this.

VI. ASPIRATIONS FOR THE FUTURE

A. Leaving aside the desire for a cure, what would you hope for in the future if you had a nephew who was diagnosed with haemophilia?

B. What advice would you give to your nephew?

C. What advice might you give to a niece who might be a carrier?
